# Current situation of allergy education in Mexico and other parts of Latin America^☆^

**DOI:** 10.1016/j.waojou.2021.100543

**Published:** 2021-05-18

**Authors:** Sandra Nora Gonzalez-Diaz, Bryan Martin, Cindy Elizabeth de Lira-Quezada, Rosalaura Virginia Villarreal-Gonzalez, Rosa Ivett Guzman-Avilan, Alejandra Macías-Weinmann, José Antonio Ortega-Martell, Carlos Macouzet-Sanchez, Mario Sánchez-Borges, Nelson Augusto Rosario Filho, Anahí Yañez, María Antonieta Guzman-Melendez, Ricardo Cardona, Olga Patricia Monge-Ortega, Ivan Cherrez-Ojeda, Dayanara Herrera-Castro, Marylin Valentin-Rostan, Juan Carlos Sisul-Alvariza, Ignacio J. Ansotegui, Barbara Elizondo-Villarreal

**Affiliations:** a Regional Center of Allergy and Clinical Immunology, University Hospital “Dr. José Eleuterio González”, Gonzalitos y Madero s/n Colonia Mitras Centro, Monterrey, NL, CP 64460, Mexico; b Allergy and Immunology, The Ohio State University, Columbus, OH, USA; c Institute of Health Sciences, Autonomous University of the State of Hidalgo (UAEH), Mexico; d Allergy and Clinical Immunology Department, Centro Médico Docente La Trinidad, Clinica El Avila, Caracas, Venezuela; e Federal University of Parana, Brazil; f Investigaciones en Alergia y Enfermedades Respiratorias - InAER, Buenos Aires, Argentina; g University of Chile, Head of Immunology and Allergy Service at the University of Chile Clinical Hospital, Chile; h Universidad de Antioquia, Colombia; i Allergology Unit of the Hospital San Juan De Dios, University of Costa Rica, San Jose, Costa Rica; j Universidad Espiritu Santo, Samborondón, Ecuador; k Hospital de Especialidades Pediátricas, Panama; l Specialist in Pediatrics, Allergy and Immunology, Montevideo, Uruguay; m Allergy and Immunology, Asuncion, Paraguay; n Department of Allergy and Immunology, Hospital Quirónsalud Bizkaia Erandio, Bilbao, Spain; o Universidad Autonoma de Nuevo Leon, Mexico

**Keywords:** Allergist, Education, Latin America, Mexico, Training

## Abstract

Allergic diseases are one of the most frequent chronic diseases in the world. It has been established that there is a worldwide epidemic of allergic diseases; therefore, the treatment of allergies should be acknowledged as a worldwide priority and the specialty of allergy should be considered an important field in medicine. Due to the fact that allergic diseases involve many organs, and Allergy and Clinical Immunology is one of the specialties in which physicians may be trained to treat patients of all ages, the subject in medical schools is not always taught as an individual specialty but often as part of another subject such as internal medicine or pediatrics.

Certified allergists are an important contribution to health systems, providing the necessary care for patients who have allergic diseases. Undergraduate programs in many universities do not include allergy as a subject, contributing to a lack of knowledge regarding the correct management of allergic diseases. World Health Organization (WHO) recommends 1 allergist per 50,000 people; however, there is an uneven distribution of allergy and clinical immunology specialists. Most practitioners are localized mainly in larger cities and state capitals, while in other regions, specialists are still greatly needed. Support and training systems are required for allergy and clinical immunology specialists to promote continuing education and keep their clinical competence up to date, which will lead to better care for their patients. Increased exposure to the concepts of allergy and clinical immunology diagnosis and treatment in undergraduate education may also potentially lead to an increase in interest in the field of allergy and clinical immunology among physicians in training. This review will approach allergy education in Mexico and other parts of Latin America.

## Introduction

Allergic disease is one of the most frequent chronic diseases in the world. It has been established that there is a worldwide epidemic of allergic diseases due in great part to modern urban lifestyle and a wide range of epigenetic modifications.^1^ Therefore, the treatment of allergies should be acknowledged as a worldwide priority, and the specialty of allergy should be considered an important field in medicine. This review will approach allergy education in Mexico and Latin America. The authors are experts in the field of Allergy and Clinical Immunology from Mexico and various areas of Latin America and have provided detailed information, according to each country that is included in this manuscript.

According to World Health Organization (WHO), there are 400 million people with allergic rhinitis and over 330 million people with asthma worldwide. In Mexico, there are 8.5 million people with asthma. In 2013, the Mexican General Health Information Committee, which coordinates and analyzes budgeting of public health and service provision, registered that 20% of hospital expenditures due to chronic respiratory diseases were because of asthma, finding an underdiagnosis and inadequate treatment for patients.^2^ This issue is not limited to Mexico, a recent European paper notes that allergic diseases are still not well recognized in all groups of ages by other specialties and government agencies.^3^

Petitions have been made to the Mexican Senate by the Global Alliance Against Chronic Respiratory Diseases (GARD) in Mexico, the Mexican College of Pediatricians Specialized in Clinical Immunology and Allergy (COMPEDIA), the Mexican College of Clinical Immunology and Allergy (CMICA) and the Latin American Society of Allergy and Immunology (SLaai), requesting that asthma and other chronic respiratory diseases be considered as an important health issue in Mexico. This recognition would create awareness and obtain greater funding for prevention and treatment. In February 2005, the Mexican Federal Journal published the first initiative to recognize asthma as a public health issue.^4^ On March 28, 2006, the second initiative presented the idea that the diagnosis and treatment of allergic diseases should be managed only by board-certified specialists in Allergy and Clinical Immunology.

Given the high prevalence of allergic diseases and the different health systems all over the world, patients might be treated by General Practitioners (GP), internal medicine specialists, pediatricians, or by professionals who have received additional training in allergy or by certified allergists. In an attempt to ensure these patients receive appropriate care, World Allergy Organization (WAO) recommendations for acceptable competence required for any physician who takes care of patients with allergy have been published.^5^

Physicians who care for patients should obtain and maintain certain medical competencies including: recognition of the essential elements of the medical profession, ethical principles and legal responsibilities; comprehension of the importance of the benefit of the patient, society and the profession, with special attention to professional confidentiality, knowledge in applying the principle of social justice to professional practice and the development of professional practice with respect to the patient's autonomy, beliefs and culture.^6^

Due to the fact that allergic diseases involve many organs and Allergy and Clinical Immunology is one of the specialties in which physicians may be trained to treat patients of all ages, the subject in medical schools is not always taught as an individual specialty but often as part of another subject such as internal medicine or pediatrics.^5^^,^^7^ It may also be taught in part, within organ based classes, where it is considered a part of Dermatology, Otolaryngology, Ophthalmology, Pulmonology and/or Gastroenterology.

It is also a relatively new discipline, and there are not yet many allergists available to teach this multidisciplinary subject. WAO describes this as the “knowledge/practice gap”.^8^ Many physicians will not qualify with adequate competency to manage the diagnosis and treatment of allergic diseases at the primary care level, unless allergy training is considered as an essential part of undergraduate medical education at the clinical level.^8^

## Allergy education

An Allergy and Clinical Immunology physician is a sub-specialist who has completed training in the area of allergy and clinical immunology after receiving education in Pediatrics or Internal Medicine. Due to the nature of allergic diseases, with a dysregulated immune response as a key feature, the study of clinical immunology is essential to understand allergy. Certified allergists are an important contribution to the health systems, providing the necessary care for patients who have allergic diseases.^9^

### Undergraduate allergy education in Mexico and other countries in Latin America

Although there are 66 medical schools in Colombia, Ramírez-Zuluaga et al found that undergraduate programs in many universities do not include allergy as a subject, but only as topics, contributing to a lack of knowledge regarding the correct management of allergic diseases.^9^ Brazil has the second largest number of medical schools in the world, with 345 schools, while Uruguay has 2 schools of Medicine, one private facility and another public one with 350 graduates every year. In Argentina there are 20 faculties of Medicine; Paraguay has 15 validated careers in Medicine; Venezuela has 8 schools of Medicine; and Chile has 20, while in Panama, there are 5 (Table 1). In Mexico, there are over 140 public and private medical schools of which only 8 states include allergy in their programs (Aguascalientes, Baja California, Chihuahua, Mexico City, Guanajuato, Jalisco, and two in Nuevo León)^10^ (Table 2).

**Table 1:** Allergy and immunology situation regarding education, training and healthcare providers in Latin America

	Certified Medical Schools Including Allergy and/or Immunology in Undergraduate Program	Training Centers for Postgraduate Allergy and Immunology Programs	Allergy Centers for Patient Care	Immunodeficiency Centers	WAO Centers of Excellence	Accreditation
Argentina	20, Allergy and Immunology is included in pediatrics or internal medicine modules.	5, Universidad de Buenos Aires -UBA. Universidad Nacional de La Plata Universidad Nacional de Córdoba Universidad Católica de Córdoba Hospital Reina Fabiola Hospital Nacional de Clinicas Córdoba	26, Hospital San Martín - Hospital Rodolfo Rossi - Hospital San Juan de Dios - Hospital Ricardo Guterrez - Hospital de niños Sor María Ludovica - Hospital Regional Mar del Plata - Hospital Penna - Hospital Provincial Centenario - Hospital Italiano Universitarios - Complejo Medico Policia Federal Argentina- Churruca Visca - Hospital de Niños Pedro Elizalde - Hospital Ramos Mejia - Hospital Rivadavia - Hospital de Niños de la Santicima Trinidad - Hospital Cordoba - Hospital Durand - Hospital Argerich - Hospital Fernandez - Hospital Alvarez - Hospital Penna - Hospital Piñero - Hospital Pirovano	16, Hospital de Pediatría Prof. Dr. Juan P Garrahan - Hospital de Niños Ricardo Gutiérrez - Hospital de Niños Pedro de Elizalde - Hospital Italiano de Buenos Aires - Hospital Británico de Buenos Aires - Hospital Nacional Prof. Alejandro Posadas - Hospital de Niños Sor María Ludovica - Hospital Universitario Austral - Hospital Rossi - Hospital Interzonal Especializado Materno Infantil (HIEMI) Victorio Tetamanti - Hospital Municipal de Agudos Dr. Leónidas Lucero - Hospital Nacional de Clínicas - Hospital Provincial de Niños de la Santísima Trinidad - Clínica Universitaria Reina Fabiola - Universidad Católica de Córdoba - Hospital Central - Hospital Pediátrico Alexander Fleming	None	CONEAU - National Commission for University Evaluation and Accreditation –Ministry of Health of the Nation
Brazil	345, Medical Schools; Allergy and Immunology is included in pediatrics or internal medicine modules.	17, Recognized training centers in Curitiba, Sao Paulo, Rio de Janeiro, Brasilia, Ribeirão Preto, Uberlândia, Salvador and Recife	Aracaju – Universidade Federal de Sergipe (UFS); Curitiba – Universidade Federal do Parana ´(UFPR); Sao Paulo – Universida de de Sao Paulo (USP)- Universidade Federal de Sao Paulo (UNIFESP); Belo Horizonte – Universidade Federal de Minas Gerais (UFMG); Campinas – Universidade Estadual de Campinas (UNICAMP); Ribeira ~ o Preto – (USP); Recife – Universidade Federal de Pernambuco (UFPE); Rio de Janeiro – Universidade Federal do Rio de Janeiro (UFRJ) and Salvador – Universidade Federal da Bahia (UFBA)	Universidad de Federal de São Paulo (UNIFESP) and Children's Institute (USP) and other 52 centers that treat PID.	Federal University of Parana (Curitiba, Brazil)	Ministry of Health of Brazil
Chile	20, Allergy topics are partially included	1, Immunology Specialist Training Program, Universidad de Chile	Private and public hospitals in Santiago and surrounding regions.	2, Clinical Hospital of the Universidad de Chile Hospital Luis Calvo Mackenna	1, Allergy, Immunology and HIV Service of the Medicine Department, Universidad de Chile	Ministry of Health of Chile
Colombia	66, Accredited by the Colombian Association of Faculties of Medicine (ASCOFAME); Allergy topics are partially included	2, Universidad de Antioquia Universidad ICESI As well as masters and doctorates in Immunology at the University of Antioquia and the University of Cartagena.	Over 40 specialists offices and services including the IPS Universitaria Clinical Allergology Service, Universidad de Antioquia - Allergology Service of the San Vicente University Foundation - Allergology Service of the Pablo Tobon Uribe Hospital - Specialized Allergy Center - Cealer Clinical Allergology Unit -Allergology Unit IPS -Alergo Clinical - Allergy Center Clinical Allergology Service Valle del Lili Foundation/Icesi University - Allergology Service at the INBANACO Clinic - Allergology service of the SantaFe foundation -Immunoallergy center in Cartagena de Indias	5, Two at the University Foundation of Health Sciences (FUCS) (Reference Center for Inborn Errors of the Immune System) University of Antioquia (Medellín) with the group of Primary Immunodeficiencies Immunoallergy Center in Cartagena de Indias and the Immunoallergy Center in Neiva	2, Group of Clinical and Experimental Allergy (GACE) (Medellin, Colombia) and the Institute for Immunological Research (Cartagena, Colombia)	Ministry of Health Republic of Colombia
Costa Rica	8, Allergy and Immunology is included in pediatrics or internal medicine modules.	None	3, Hospital San Juan de Dios Hospital Mexico Hospital de Niños	3, Hospital San Juan de Dios Hospital Mexico Hospital de Niños	None	Ministry of Health, Costa Rican Social Security
Ecuador	23, and 12 of them include Allergy as a subject	None	14, Hospital Kennedy Hospital Alcivar Hospital Interhospital Hospital de niños Roberto Gilbert Hospital Teodoro Maldonado Carbo. Hospital Del Rio Hospital Seguro Social de CuencaHospital Monte Sinai Hospital Carlos Andrade Marin Hospital Quito N.1 de la Policia Nacional Hospital de Especialidades Portoviejo Hospital Seguro Social PortoviejoHospital de los Valles QuitoHospital del Seguro Social Machala	PIDE Foundation (Patients with Primary Immunodeficiency Ecuador)	None	Ministry of Health of Ecuador
Panama	5, Allergy and Immunology is included in internal medicine modules.	1, Arnulfo Arias Madrid Complex	5, Complejo Hospitalario Arnulfo Arias Madrid Hospital de Especialidades Pediátricas Omar Torrijos Herrera Hospital del Niño Hospital Chicho Fábrega en Santiago de Veraguas Hospital José Domingo De Obaldía	5, Complejo Hospitalario Arnulfo Arias Madrid Hospital de Especialidades Pediátricas Omar Torrijos Herrera Hospital del Niño Hospital Chicho Fábrega en Santiago de Veraguas Hospital José Domingo De Obaldía	None	Ministry of Health of Panama
Paraguay	15 nationally credited Faculties of Medicine and 3 under the model Mercosur/Regional International. The Universidad Nacional de Asuncion includes allergy topics as part of the subjects: Clinical Medicine and Pediatrics.	2, Universidad Nacional de Asuncion through the Social Prevision Institute Universidad Catolica in Asuncion	6, Universidad Nacional de AsuncionInstituto de Prevision SocialHospital Nacional de Itagua Clinica Guggiari Clinica Sisu in Asuncion and Villarrica	1, Department of Immunology at the Institute of Health Research Sciences of the Universidad Nacional de Asuncion	None	Postgraduate schools of the national universitiesMinistry of Health and Social Welfare
Uruguay	2, Allergy topics are partially included	1, Facultad de Medicina de Uruguay, Clinical Hospital of Montevideo	5, Hospital de ClinicasHospital de Pediatria Pereira Rossell Private Clinics in Montevideo	1, Hospital Pereira Rosell	None	Ministry of Health of Uruguay
Venezuela	8, Allergy topics are included	2, Institute of Immunology of the Faculty of Medicine of the Central University of Venezuela.Allergology Service of the Central Hospital of the Armed Forces Dr. Carlos Arvelo. University residence	4, Institute of Immunology of the Faculty of Medicine of the Central University of Venezuela. - Allergology Service of the Central Hospital of the Armed Forces Dr. Carlos Arvelo. University residence - National Center of Dermatology and Allergy of the Venezuelan Institute of Social Security - The Allergy and Immunology Department of the La Trinidad Teaching Medical Center, in Caracas.	1, The Institute of Immunology of the Faculty of Medicine of the Central University of Venezuela	None	Hospitals and national medical boardsVenezuelan Society of Allergy, Asma and Immunology

**Table 2:** Allergy and immunology education, training and patient care in Mexico.

	Certified Medical Schools Including Allergy and/or Immunology in Undergraduate Program	Training Centers for Postgraduate Allergy and Immunology Programs (Allergy and Immunodeficiency Patient Care)	WAO Centers of Excellence	Accreditation
México	- Aguascalientes: 2 - Baja California: 7 - Campeche: 1 - Chiapas: 6 - Chihuahua: 3 - Ciudad de México: 14 - Coahuila: 2 - Colima: 1 - Durango: 4 - Estado de México: 4 - Guanajuato: 3 - Guerrero: 2 - Hidalgo: 1 - Jalisco: 9 - Michoacán: 2 - Morelos: 2 - Nayarit: 2 - Nuevo León: 4 - Oaxaca: 3 - Puebla: 5 - Querétaro: 3 - Quitana Roo: 2 - San Luis Potosí: 1 - Sinaloa: 1 - Sonora: 1 - Tabasco: 4 - Tamaulipas: 10 - Tlaxcala: 1 - Veracruz: 8 - Yucatán: 3 - Zacatecas: 1	15 - Antiguo Hospital Civil de Guadalajara Fray Antonio Alcalde - Centro Regional de Alergia e Inmunología Clínica del Hospital Universitario “Dr. José Eleuterio González”^a^ - Centro Médico Nacional 20 de Noviembre - Centro Médico Nacional Siglo XXI, Instituto Mexicano del Seguro Social (IMSS) - Centro Médico Nacional de Occidente, Instituto Mexicano del Seguro Social (IMSS) - Hospital General de México “Dr. Eduardo Liceaga” - Hospital Infantil de México Federico Gómez - Hospital Universitario de Puebla^a^ - Hospital Regional Lic. Adolfo López Mateos - Hospital Regional “Dr. Valentín Gómez Farías” - Hospital General Centro Médico Nacional La Raza, Instituto Mexicano del Seguro Social (IMSS) - Hospital de Pediatria Centro Médico Nacional de Occidente, Instituto Mexicano del Seguro Social (IMSS) - Instituto Nacional de Enfermedades Respiratorias (INER) - Instituto Nacional de Pediatría (INP) - Hospital Juárez de México	4 COE 2016: Centro Regional de Alergia e Inmunología Clínica del Hospital Universitario “Dr. José Eleuterio González” 2016: Hospital Infantil de México Federico Gómez 2018: Centro Médico Nacional Siglo XXI 2019: Centro Médico Nacional 20 de Noviembre	CONICA (National Council of Clinical Immunology and Allergy)

COE: Centers of Excellence.

Asociación Mexicana de Facultades y Escuelas de Medicina, A.C. (AMFEM). Directory of Affiliated Faculties and Schools. (January 20, 2021). https://www.amfem.edu.mx.

World Allergy Organization. WAO Centers of Excellence. Latin America. (January 20, 2021). https://www.worldallergy.org.

Consejo Nacional de Inmunología Clínica y Alergia (CONICA). (March 10, 2021). http://www.conica.org.mx.

a National Program of Quality Postgraduates. International Competence of the National Council of Science and Technology. (Conacyt, July 2020)

In the rest of the world, teaching of allergy as a subject is not always homologous and is taught in academic postgraduate programs as follows: in 16 countries as respiratory medicine, 15 as part of pediatrics, 12 countries include it as part of dermatology, 14 in otolaryngology, 14 as part of internal medicine, 1 country includes it in gastroenterology, and only in 1 country is allergy part of the training of the General Practitioner.^11^ It is important that undergraduate students are exposed to the specialty in order to promote their interest and enthusiasm towards becoming specialists in the future. In the same way, this may happen with pediatric residents since the time period spent in allergy training is often not long.^3^^,^^12^

### Postgraduate allergy education in Mexico and other countries in Latin America

In Latin American countries, allergy training is available in Argentina, Brazil, Chile, Colombia, Mexico, Paraguay, Peru, Dominican Republic, Uruguay, and Venezuela. Allergology is considered a third-level patient care specialty or subspecialty in 56% of Latin American countries.^13^

Specialized referral hospitals include third level care specialties, where allergy and immunology centers provide patient care. In the case of subspecialties, 2–3 years of training is needed in internal medicine or pediatrics before physicians can access allergology.^13^

Allergology training is found as second-level medical care in some countries. No basic specialization is needed and training is accessible for a general physician. General Practitioners in Brazil can start a residency after practicing allergology for at least 8 years, and result with a university degree. Children and adult care is included in most training centers. The Brazilian Association of Allergy and Immunopathology has training programs in 17 accredited centers, associated mainly with medical schools. A 2-year residency in Allergy and Clinical Immunology requires 2 years of previous training in Internal Medicine. Fellowship programs are offered for pediatricians, which require at least 2-years of training in pediatrics. The Brazilian Group of Primary Immunodeficiencies (BRAGID) was created in 2001 aiming to improve primary immunodeficiencies (PID) diagnosis through educational activities and to stimulate research in PID. This group established educational activities and stimulated research in PID. BRAGID has been linked to the Latin American Group of Primary Immunodeficiencies (www.lasid.org) which in turn became a society in 2009. The BRAGID website has more than 4200 members; among them 41% are pediatricians. The educational task on early suspicion of PID is a challenge with targets such as the warning signs, for medical students and all health professionals.^14^

Brazil and Mexico have a special training program with a university title for pediatric allergists. Colombia and Venezuela each have 2 training centers with 4 residents per year in Allergy and Clinical Immunology. In Argentina, 5 universities including the Universidad de Buenos Aires, Universidad Nacional de La Plata, Universidad Nacional de Córdoba, and the Universidad Católica de Córdoba, all have Allergy and Immunology training programs which are accredited by the Argentine Association of Allergy and Clinical Immunology (AAAeIC) as an advanced specialist course with a duration of 2–3 years as well as by the Ministry of Health.^15^ Ecuador and Costa Rica have no residencies for allergology, while in Panama there is 1 training center. After receiving their university title, allergology candidates are required to be evaluated by a national board before practicing allergology.^13^

Generally, no new examinations take place; however, recertification is required every 2–5 years. In countries such as Paraguay, certification is achieved by attending courses and congresses from the start, and physicians are not required to be certified through examination. Postgraduate schools of national universities as well as the Ministry of Health and Social Welfare in Paraguay authorize medicine and allergy practice before physicians start evaluating patients.^13^

For the most part, the basic curricula in pediatric or internal medicine as well as third level allergology training has a duration of 4–6 years. Three years is the duration in places where no primary training is required before second-level allergology completion.^13^ Recently, postgraduate courses have been designed for all levels of healthcare professionals who encounter patients with allergic conditions, including doctors, specialist nurses, and dietitians.

Some faculties have distance learning options available. The courses may be run by leading world experts and incorporate up-to-the-minute information on new research and clinical practice.

In Mexico there are currently 15 training centers (Table 2) with the purpose to educate specialists in Allergy and Clinical immunology to be capable of providing medical care for patients with allergic and immunologic diseases; they have abilities, skills, and aptitude in diagnosis, treatment, and prevention. The subspecialty is 2 years long and in order to apply the physician must have his or her degree in Pediatrics or Internal Medicine.

### Skills of the allergist^16^

• Understanding the development and mechanism of allergic and immunologic diseases
• Comprehension of the concept “one airway, one disease” as well as the "atopic march"
• Effective communication with young patients and their parents/caregivers
• Physical exam of patients
• Education towards anaphylaxis prevention.
• Education including multidisciplinary treatment in atopic dermatitis/eczema
• Correct use and adequate training techniques of inhalers in patients with asthma
• Diagnosis and treatment of food allergy as well as venom allergy
• Comprehension and understanding of primary and secondary prevention in asthma and allergic rhinitis
• Understanding the benefits regarding treatment of allergic diseases
• Comprehension and understanding about primary immunodeficiencies
• Understanding the benefits of early diagnosis and opportune referral for treatment in primary immunodeficiencies and autoimmune diseases

WHO recommends 1 allergist per 50,000 people; however in Mexico the ratio of allergists per population is 1:175 000 persons.^17^ Currently, there are 1115 specialists in allergy and clinical immunology in Mexico, of which 68.3% are certified by the National Board of Clinical Immunology and Allergy (CONICA) and it is necessary to renew the certification every 5 years by collecting 250 points, in accordance with the guidelines of the National Standards Committee of Medical Specialty Boards (CONACEM) and the Medical Specialty Boards according to the 81st article of the General Law of Health, based on the 272 BIS article and the fourth title.^18^ In Mexico, 54.5% of allergists are women.^19^

In Mexico, there is an uneven distribution of allergy and clinical immunology specialists. Most practitioners are localized mainly in larger cities and state capitals, while in other regions specialists are still greatly needed.

In 1946, the Mexican Society of Allergy was created in Tampico, Tamaulipas, Mexico. The founding members include Dr. Carlos Canseco, Dr. Julio Cueva, Dr. Arturo Blackaller, Dr. Mario Salazar Mallén, Dr. Oscar de la Fuente, and Dr. Luis Cortés. This society later took the name of the Mexican College of Clinical Immunology and Allergy A.C. (CMICA), which is what governs to this day. The Mexican College of Pediatricians Specialized in Clinical Immunology and Allergy (COMPEDIA) was founded in 1981 by Dr. José G. Huerta López, in the city of Puebla. The Mexican Journal of Allergy (Revista Alergia México) that has published continuously since 1953 and the Journal of Pediatric Allergy (Revista de Alergia Pediátrica) are the 2 main journals in the allergy field in Mexico.^14^

In the last decades, the Mexican and Latin American Allergy communities have been greatly involved in WAO, becoming leaders in allergy and immunology education. Their high standards in research, education, and community assistance have made them eligible to be included as WAO Centers of Excellence.

### World Allergy Organization Centers of Excellence

A WAO Center of Excellence is identified as a multidisciplinary group, based in an academic institution, encompassing 3 or more subspecialties with the following components: graduate programs with academics and students, peer-reviewed publications in the field of allergy and immunology, well-established organization with good governance, infrastructure, recognized mentors in allergy, asthma, and clinical immunology, and a research department.

**Fig. 1:**
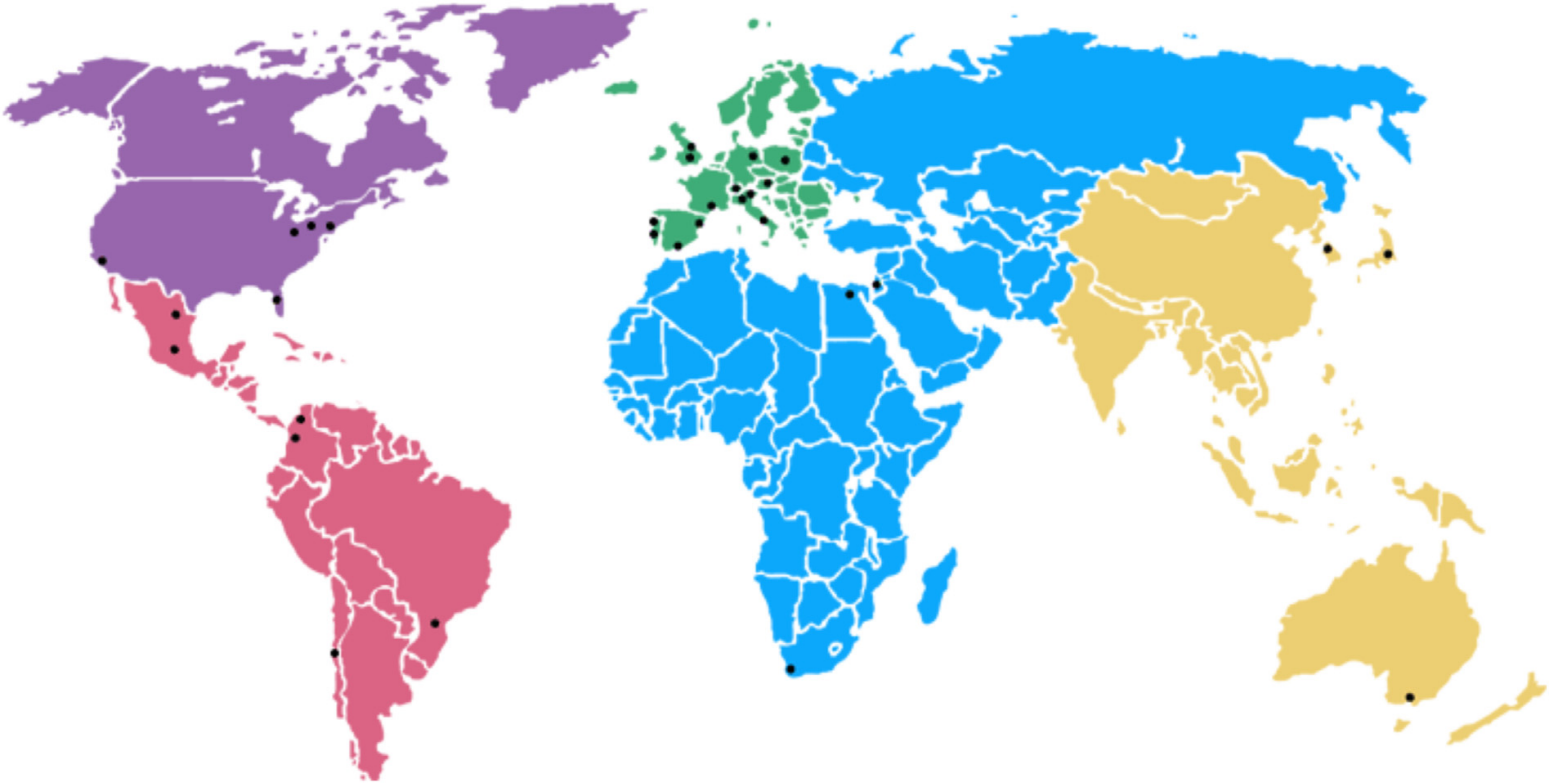
WAO centers of excellence

A WAO Center of Excellence should be proficient in the following areas: research, basic and clinical science, and recognized education and training program (Fig. 1).

The following information was obtained at WAO Centers of Excellence [Internet]. WAO World Allergy Organization. 2020 [cited July 20, 2020]. Available at https://www.worldallergy.org In Latin America there are 8 Centers of Excellence^20^:

1. Centro Regional de Alergia e Inmunología Clínica (CRAIC) (Monterrey, Mexico): 2016 Center of Excellence

Undergraduate and postgraduate training center providing prevention-oriented health care services in the outpatient clinic and research center with high-quality standards, in benefit of the general community of Monterrey and other surrounding states in northeastern Mexico. CRAIC is located in a total area of 1000 m^2^ on the fourth floor of the outpatient clinic building in the UANL's University Hospital. Medical services are primarily focused on prevention, treatment, and rehabilitation of immuno-allergic diseases through comprehensive patient care for adults and children specializing in respiratory, skin, drugs, food, and hymenoptera allergy as well in immunodeficiency disorders.

Since 2000, Sandra Nora Gonzalez-Diaz, MD PhD, has led CRAIC and encouraged a culture of international collaboration in teaching, clinical medicine, and research, alongside the staff of professors.

2. Centro Medico Nacional Siglo XXI- Instituto Mexicano del Seguro Social (Mexico City, Mexico): 2016 Center of Excellence

The Allergy and Clinical Immunology service of the Hospital de Especialidades, Centro Medico Nacional Siglo XXI, in Mexico City, belongs to the largest public social security institution in the country. It is a national reference center for diagnosis and specialized treatment in allergic diseases and immunodeficiencies. Their mission is to be a service equipped with high technology, resolutive capacity, and constant training, which meets the needs and expectations of the right-holders, contributing with quality scientific research and high-level educational training for our resident doctors, forging the best Allergy and Clinical Immunology service in the country.

Since 2015, it has been led by Nora Hilda Segura Méndez MD, who also serves as editor-in-chief of the Revista Alergia México, which is recognized as the most important scientific journal of the specialty in Latin America. She is the head professor of the graduate course in Allergy and Clinical Immunology. The center has national and international publications, and the main lines of research are primary immunodeficiencies, anaphylaxis, and asthma.^20^

3. Clinical Hospital University of Chile (Santiago, Chile): 2019 Center of Excellence

Their service was the first of its kind in Chile, and was founded by Professor Cecilia Sepúlveda MD in 1984. Later, Professor Maria Antonieta Guzman MD, current Head of the Service, founded in 2002 the Allergy Center with different levels of complexity, and then, in 2007, Professor Alejandro Afani MD, founded the HIV Center. All this work is solidly supported by 2 laboratories in the Service: Immunology and Molecular Medicine.

This Division of Immunology, Allergy and HIV is part of the Clinical Hospital of the University of Chile, the main training center for specialists in the country. The professional team includes 10 MD specialists in Clinical Immunology, two Biochemists (one of them with a PhD degree), 11 medical technologists and 1 psychologist with a PhD degree.

4. Federal University of Parana (Curitiba, Brazil): 2018 Center of Excellence

Nelson A. Rosario MD, PhD, FACAAI is currently the Chairman of the Division of Pediatric Allergy, Immunology and Pneumology in the Hospital de Clinicas. He has trained over 40 allergy specialists. The Federal University of Paraná (*Universidade Federal do Paraná*, UFPR) is a public university headquartered in Curitiba, Paraná, Brazil. UFPR ranks as 37th best university in Latin-America and it is among the 651–700 best universities in the world, according to QS World University Rankings. It is placed as the 8th best university in Brazil in the latest “Ranking Universitário Folha (RUF)", published by the nation's largest newspaper.^21^

Nowadays, its facilities are spread over the capital Curitiba and other cities of the State of Paraná. It offers 124 undergraduate degree courses, 44 doctorate, 66 masters, and 5 professional masters programs.^22^

5. Group of Clinical and Experimental Allergy (GACE) (Medellin, Colombia): 2016 Center of Excellence

The Research Group in Clinical and Experimental Allergy (GACE) was created in 2007, thanks to the support of the Faculty of Medicine and the School of Microbiology at the University of Antioquia, to strengthen, generate, and promote research, and disseminate knowledge in clinical allergy, basic areas and translational research, to scientist and students through training and formation. Their aim is to establish appropriate conditions for conducting research, teaching and extension processes in the field of clinical allergy, epidemiology of allergic diseases, and in experimental allergology (molecular, genetics and applied biotechnology), to contribute to the research culture and knowledge management.^20^ They have an excellent team lead by Ricardo Cardona Villa (MD, MSc), composed of teachers and researchers, professionals, graduate and undergraduate students who are part of research projects and clinical practice.^20^

6. Hospital Infantil de Mexico Federico Gomez (Mexico City, Mexico): 2016 Center of Excellence

The Allergy, Asthma and Clinical Immunology service is a regional Comprehensive Care Center for the evaluation, diagnosis and treatment of patients with suspected allergic diseases, difficult asthma and immunodeficiency disorders. The mission of the Center is to give medical attention of high specialty on pediatric allergy and clinical immunology, giving safe attention and a high quality by sub-specialist on pediatrics, and doctors in training, and also to carry out excellence scientific research on allergic and immunologic diseases and their comorbidities.^20^

They have an excellent team lead by Dr. Blanca E. Del Rio Navarro composed of teachers and researchers, professionals, graduate and undergraduate students.^19^

7. Institute for Immunological Research (Cartagena, Colombia): 2016 Center of Excellence

The Institute for Immunological Research of the University of Cartagena is devoted to research and education in Clinical and Experimental Allergy. It has 2 graduate programs (MSc/Immunology and PhD/Biomedical Sciences) and supports the training in basic research of fellows in training from the Department of Pediatrics of the University of Cartagena, School of Medicine. Enrolled in these programs there are currently 22 PhD, 6 MSc, and 8 undergraduate students. In addition, the Institute for Immunological Research of the University of Cartagena has 4 undergraduate programs in Basic and Clinical Immunology for the Schools of Medicine, Dentistry, Pharmacy, and Nursing.

The Institute for Immunological Research of the University of Cartagena is led by Professor Luis Caraballo MD, PhD and has a high quality scientific and academic staff. As a joint effort among national and foreign colleagues, well trained technicians, PhD and MSc students as well as specialty trainees and undergraduate students, the work of the Institute for Immunological Research of the University of Cartagena covers several fields of Basic Immunology, Molecular Genetics, Biotechnology, Experimental Allergy, Clinical Allergy, Epidemiology, Pediatrics and ENT.^19^

8. National Medical Center “20 de Noviembre” (Mexico City, Mexico): 2019 Center of Excellence

The center was opened in May 1961, as the “Centro Hospitalario 20 de Noviembre”. This clinic was initially led by Manuel Romero Herrera M.D and Jorge Guillen Toledo M.D.; in 1980 Guillen Toledo M.D. took on the position of head of the clinic, which had by that time changed the name to “Service of Allergy and Clinical Immunology".

Since 1979, specialists of Allergy and Clinical Immunology have been trained at this center and in 1985, thanks to the quality of the service, the teaching and the facilities, the Universidad Nacional Autónoma de México (UNAM) recognized the formation of specialists in our discipline. Since March 2010, Dr. María Eugenia Vargas Camaño has overseen postgraduate courses and Dr. María Isabel Castrejón Vazquez has worked in this clinic, with 4 physicians in training as Allergy and Clinical Immunologists.^20^

## Conclusions

Allergic diseases are a major global health problem, also considered one of the epidemics of the XXI century. Despite the growing number of patients suffering from atopic disorders, there is still a deficiency of specialists trained in the diagnosis, treatment and prevention of this group of diseases. The undergraduate programs of a large number of universities in Mexico and other Latin American countries do not include an adequate curriculum in allergy, which contributes to the lack of information and the incorrect management of patients with increasingly common atopic diseases.

The standards in which it is determined that a physician specializes in allergy and clinical immunology are important because allergic diseases are very common. The availability of services and training for allergists is disproportionate, with a great need not yet covered. Support and training systems are also required for allergists and clinical immunologists to promote continuing education and keep their clinical competence up to date, which will lead to better care for their patients. Increased exposure to the concepts of allergy and clinical immunology diagnosis and treatment in undergraduate education may also potentially lead to an increase in interest in the field of allergy and immunology among physicians in training.

## Abbreviations

CONACEM: National Standards Committee of Medical Specialty Boards, CONICA: National Board of Clinical Immunology and Allergy, COMPEDIA: Mexican College of Pediatricians Specialized in Clinical Immunology and Allergy, CMICA: Mexican College of Clinical Immunology and Allergy A.C. ENT: Ears, nose and throat, GACE: Research Group in Clinical and Experimental Allergy, GARD: Global Alliance Against Chronic Respiratory Diseases, GP: General practitioner, MD: Doctor of Medicine, MSc: Master of Science, PhD: Doctor of Philosophy, SLAAI: The Latin American Society of Allergy and Immunology, UANL: Universidad Autónoma de Nuevo León, UNAM: Universidad Nacional Autónoma de México.

## Funding

The authors declare that no funding was received for the present study.

## Author contributions

Sandra Nora Gonzalez-Diaz: Design of the study, manuscript elaboration and revision.

Bryan Martin: Design of the study, manuscript elaboration and revision.

Cindy Elizabeth de Lira-Quezada: Design of the study, manuscript elaboration and revision.

Rosalaura Virginia Villarreal-Gonzalez: Design of the study, manuscript elaboration and revision.

José Antonio Ortega Martell: Design of the study, manuscript elaboration and revision.

Rosa Ivett Guzman Avilan: Manuscript elaboration.

Alejandra Macías-Weinmann: Manuscript elaboration.

Carlos Macouzet-Sanchez: Manuscript elaboration.

Mario Sánchez-Borges: Manuscript elaboration.

Nelson Rosario: Manuscript elaboration.

Anahí Yañez: Manuscript elaboration.

María Antonieta Guzman: Manuscript elaboration.

Ricardo Cardona: Manuscript elaboration;

Olga Patricia Monge Ortega: Manuscript elaboration;

Dayanara Herrera-Castro: Manuscript elaboration.

Ivan Cherrez-Ojeda: Manuscript elaboration.

Marylin Valentin-Rostan: Manuscript elaboration.

Juan Carlos Sisul: Manuscript elaboration.

Ignacio Ansotegui: Manuscript elaboration.

Barbara Elizondo: Manuscript elaboration.

## Declaration of competing interest

The authors declare they have no conflicts of interest to disclose.
